# Molecular identification of nontuberculous mycobacteria isolated from pyogenic bovine tissues in South Darfur State and Alsabalouga slaughterhouse at Omdurman area, Sudan

**Published:** 2014-02-25

**Authors:** A.E. El Tigani-Asil, S.M. EL Sanousi, M.A. Aljameel, H. El Beir, A. Adam, M.M. Abdallatif, M.E. Hamid

**Affiliations:** 1 *Departments of Veterinary Medicine, Faculty of Agriculture and Veterinary Medicine, Qassim University, Saudi Arabia*; 2 *Departments of Microbiology, Faculty of Veterinary Medicine, University of Khartoum, Sudan*; 3 *Department of Pathology, Veterinary Research laboratory - Nyala, National Veterinary Research Corporation, Sudan*; 4 *Department of Tuberculosis, Institute of Tropical Diseases, Ministry of Science and Technology, Sudan*; 5 *Department of Pathology, Faculty of Veterinary Science, University of Nyala, Sudan*; 6 *Department of Microbiology, Faculty of Science, North Border University, Saudi Arabia*; 7 *Department of Clinical Microbiology and Parasitology, College of Medicine, King Khalid University, Saudi Arabia*

**Keywords:** Acid fast bacteria, Bovine farcy, Nontuberculous mycobacteria, Pyogenic infection

## Abstract

This study identified nontuberculous mycobacteria (NTM) recovered from bovine pyogenic affections obtained at necropsy using the molecular target 16S-23S rDNA internal transcribed spacer region. Postmortem inspection of cattle was conducted at South Darfur State abattoirs and Alsabalouga Slaughterhouse at Omdurman area during 2007-2009. Specimens were examined for the presence of acid fast bacteria (AFB) using microscopic and standard culturing techniques. AFB were identified phenotypically and confirmed by 16S-23S rDNA ITS. Fifty nine NTM were recovered and confirmed as acid fast filaments out of 165 positive AFB specimens, of which 52 isolates were identified as bovine farcy causative agents, while 7 cultures were excluded due to drying. 16S-23S rDNA ITS of NTM revealed three different amplicons 500 bp. (32) isolates, 550 bp. (2) isolates and 600 bp. (14) isolates. Four isolates were contaminated.

## Introduction

The genus *Mycobacterium* is represented by a wide range of organisms found both in clinical or environmental material, with complex phenotypic and genetic data, and associated with various human and animal diseases (Shinnick and Good, 1994; Wallace, 1994). Nontuberculous mycobacteria (NTM) have emerged as public health hazards in developing countries where lack of nutrition, civil wars and environmental crisis occur (Mokaddas and Ahmad, 2007). In tropical climates NTM are frequently isolated from diverse environmental sources, many animals and human conditions (Kim *et al.*, 2005). For example, *M. farcinogenes* and *M. sengelenses* have been reported causing a chronic infection in zebu cattle namely bovine farcy (BF) (Lechevalier *et al.*, 1971; El Sanousi *et al.*, 1977). BF is an endemic non treatable disease in Africa causes lymphatic and pulmonary suppurative forms resembled to tuberculosis lesions and lead to carcasses condemnations (Chamoiseau, 1979; Mohan, 1985).

The prevalence rate of the disease in the Sudan was found as 15% in western Sudan (El Nasri, 1961), 5.19% in Nyala, 4.19% in Nuba Mountains and 3.49% in Omdurman area (El Sanousi *et al.*, 1977, 1979), while EL Tigani-Asil *et al*. (2013) reported the prevalence rate of BF as 2.2% in South Darfur State. Human infections include *M. farcinogenes* in hip joint arthroblasty (Wong *et al.*, 2005) and *M. Sengelenses* in catheter-related bloodstream infection in a Korean cancer patient (Oh *et al.*, 2005) were reported. *M. avium* was isolated from a Friesian cross breed suffering from parotid and maxillary nodular infections similar to BF lesions, which made a clinical diagnosis difficult (Hamid *et al.*, 2007).

Phenotypic identification of mycobacteria grown in culture utilizes biochemical features (Kent and Kubica, 1985) is time-consuming and does not identify closely related taxa (Springer *et al.*, 1996). 16S-23S ribosomal DNA internal transcribed spacers (16S-23S rDNA ITS) have been considered an alternative method for identification (Roth *et al.*, 2000).

PCR amplification with primers Ec16S.1390p and Mb23S.44n resulted in the detection of a single band of approximately 480 bp in all 60 *Mycobacterium* strains investigated. The variation in product length was not considerable between the different species of slow growers, but a smaller product of approximately 430 bp was noted for *M. xenopi* strain (Roth *et al.*, 1998). This study aimed to identify NTM from bovine suppurative tissues by the genus specific 16S-23S rDNA ITS gene.

## Materials and Methods

### Test strains

Fifty two isolates were included in this study and they were phenotypically identified as farcy agents out of 59 confirmed acid fast filamentous cultures. The part of the conventional Mycobacteriology of this work was published by EL Tigani-Asil *et al*. (2012, 2013).

### References strains

*M. farcinogenes* (M39), previously typed by Hamid *et al*. (2002), was used as a positive control for typing BF agents following 16S-23S rDNA ITS method described by Roth *et al*. (1998). *M. bovis* (M116), characterized by Salih *et al*. (2005) was also used for comparison with the NTM PCR products.

### DNA extraction

A loop-full of mycobacterial colonies was suspended in 200-400 μl of 1xTE (10 mM Tris–HCl, 1 mM EDTA, pH 8.0). The suspension was boiled for 10min, and briefly centrifuged; the supernatant was used as a DNA template (Bakashi *et al.*, 2005; Taylor *et al.*, 2007; Rodríguez *et al.*, 2010).

### DNA amplification

PCR chemicals and primers were purchased from Bioneer (Bioneer Co., Daejeon, South Korea). A Master Mix of 25 μl reaction volume was prepared from 15.7 μl double distilled water, 2.5 μl PCR buffer, 1 μl (MgCl2, dNTPS, and 16S-23S rDNA primers: forward Ec 16S.1390p (5- TTG TAC ACA CCG CCC GTC A-3) and reverse 23S.44n (5- TCT CGA TGC CAA GGC ATC CAC C- 3), 0.3 μl TAQ (1 IU) and 2.5 μl DNA template. PCR condition consisted of denaturation for 5 min. at 95 ºC followed by 38 cycles of: denaturation 94 ºC, annealing at 62 ºC and extension at 72 ºC for 1 min. (Roth *et al.*, 1998; Mokaddas and Ahmad, 2007).

### Agarose Gel Preparation

Amount of 1.5g of agarose (Pharmacia Biotech AB, Uppsala, Sweden) was added to the mixture of 5ml of 10X TBE (0.023 M. Tris -Borate, 0.5 mM EDTA) buffer and 45 ml of distilled water, the mixture was melted in microwave oven for 2-5minutes, after cooling gel down to 60-70 ºC, 3 μl of ethidium bromide (0.5mg/ml) was added then the 50 ml of molten cooled agarose was loaded the 50ml gel cast (DNA plus, USA Scientific Int., Ocala, FL).

### Electrophoresis and Gel Visualization

PCR products were electrophoresed in a 1.5% agarose gel and visualized using a UV source and compared to a molecular marker 100 bp. (Bioneer Co., Daejeon, South Korea).

## Results

**Fig. 1:**
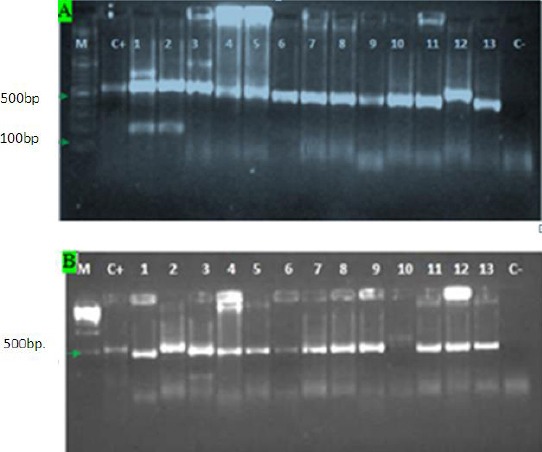
Ethidium bromide-stained agarose gel (1.5%) showing PCR amplicons of 16S-23S rDNA. Lane M= 100bp DNA ladder, C+ = *M. farcinogenes* reference strain (M39), C- = negative control. In picture A, Lane 12= *M. bovis* BCG strain (M116). (A) Lanes 1-11, 13 represented *M. farcinogenes* field strains: 9, 17, 18, 34, 35, 36, 37, 43, 75, 136, 140 and 162), respectively. (B) Lanes1-13 represented *M. farcinogenes* field strains: 244, 245, 246, 260, 264, 265, 266, 267, 270, 271, 274, 277, and 287, respectively.

Of the 52 strains included in the study, 48 electrophoresed at 500-600 bp. bands (48 isolates) when compared with *M. farcinogenes* M39 and *M. bovis* reference strains. Thirty two isolates (MF, 2, 13, 14, 36, 37, 38, 39, 43, 75, 136, 140, 162, 202, 204, 208, 216, 217, 219, 225, 244, 245, 246, 260, 264, 265, 266, 267, 270, 271, 274, 277, 287) electrophoresed at 500 bp., 2 isolates (MF, 34, 35) at 550 bp. and 14 isolates (MF, 1, 4, 9, 17, 18, 252, 288, 304, 305, 310, 314, 319, 320, 323.) at 600 bp. respectively. Four isolates were contaminated (MF, 284, 306, 321, 322) Fig. (1).

## Discussion

Until 15 years ago, species differentiation of mycobacteria was difficult especially for *M. bovis*. Identification of mycobacteria with the conventional phenotypic tools is time consuming and identification of closely related taxa is not always reproducible in contrast to molecular techniques (Roth *et al.*, 2000). Nevertheless, conventional methods still have value in developing countries where modern laboratory tools are not available. PCR is a sensitive and specific tool in diagnosis of such fastidious organisms and reduces time for identification and perhaps the cost.

In this study, amplification of a genus specific gene; 16S-23S rDNA ITS produced 500-600 bp. bands from 48 field isolates, *M. farcinogenes* (M39) and *M. bovis* (M116).

However, in previous study the same gene amplified about 480bp. from rapid grower *Mycobacterium* other than farcy agents as reported by Roth *et al*. (1998). Strains (MF: 2, 13, 14, 36, 37, 38, 39, 43, 75, 136, 140, 162, 202, 204, 208, 216, 217, 219, 225, 244, 245, 246, 260, 264, 265, 266, 267, 270, 271, 274, 277 and 287) produced 500 bp. while, strains (MF:1, 4, 9, 17, 18, 252, 288, 304,305,310,314, 319, 320 and 323) produced about 600 bp. and strains (MF: 34 and 35) revealed 550 bp.

Some strains amplicons showed double band of 500/200 (MF246), 600/200 (MF: 9, 17) and 600/350 bp (MF319) length in contrast to Roth *et al*. (1998). The variation in product length was not considerable between the different species of slow growers, but a smaller product of approximately 430 bp was noted for *M. xenopi* as reported by Roth *et al*. (1998).

16S-23S rDNA PCR analysis produced several different sized amplicons; 500-600 bp. NTM and 600bp. of *M. bovis* strain. Sequencing of PCR products of NTM is needed to clarify the different lengths of NTM obtained in this study with other possible phylogenetic analysis methods.
